# Functional and Pathogenic Differences of Th1 and Th17 Cells in Experimental Autoimmune Encephalomyelitis

**DOI:** 10.1371/journal.pone.0015531

**Published:** 2010-11-29

**Authors:** Helena S. Domingues, Marsilius Mues, Hans Lassmann, Hartmut Wekerle, Gurumoorthy Krishnamoorthy

**Affiliations:** 1 Department of Neuroimmunology, Max Planck Institute of Neurobiology, Martinsried, Germany; 2 PhD Program in Experimental Biology and Biomedicine, Center for Neuroscience and Cell Biology, University of Coimbra, Coimbra, Portugal; 3 Center for Brain Research, Medical University of Vienna, Vienna, Austria; New York University, United States of America

## Abstract

**Background:**

There is consensus that experimental autoimmune encephalomyelitis (EAE) can be mediated by myelin specific T cells of Th1 as well as of Th17 phenotype, but the contribution of either subset to the pathogenic process has remained controversial. In this report, we compare functional differences and pathogenic potential of “monoclonal” T cell lines that recognize myelin oligodendrocyte glycoprotein (MOG) with the same transgenic TCR but are distinguished by an IFN-γ producing Th1-like and IL-17 producing Th17-like cytokine signature.

**Methods and Findings:**

CD4^+^ T cell lines were derived from the transgenic mouse strain 2D2, which expresses a TCR recognizing MOG peptide 35–55 in the context of I-A^b^. Adoptive transfer of Th1 cells into lymphopenic (Rag2^−/−^) recipients, predominantly induced “classic” paralytic EAE, whereas Th17 cells mediated “atypical” ataxic EAE in approximately 50% of the recipient animals. Combination of Th1 and Th17 cells potentiated the encephalitogenicity inducing classical EAE exclusively. Th1 and Th17 mediated EAE lesions differed in their composition but not in their localization within the CNS. While Th1 lesions contained IFN-γ, but no IL-17 producing T cells, the T cells in Th17 lesions showed plasticity, substantially converting to IFN-γ producing Th1-like cells. Th1 and Th17 cells differed drastically by their lytic potential. Th1 but not Th17 cells lysed autoantigen presenting astrocytes and fibroblasts *in vitro* in a contact-dependent manner. In contrast, Th17 cells acquired cytotoxic potential only after antigenic stimulation and conversion to IFN-γ producing Th1 phenotype.

**Conclusions:**

Our data demonstrate that both Th1 and Th17 lineages possess the ability to induce CNS autoimmunity but can function with complementary as well as differential pathogenic mechanisms. We propose that Th17-like cells producing IL-17 are required for the generation of atypical EAE whereas IFN-γ producing Th1 cells induce classical EAE.

## Introduction

Experimental Autoimmune Encephalomyelitis (EAE), an animal model representing human multiple sclerosis (MS), is mediated by CD4^+^ helper T cells which trigger an (auto)-inflammatory response against central nervous system (CNS) structures that culminates in demyelination, axonal damage and paralysis. Over decades, IFN-γ secreting Th1 cells primed by a heterodimeric cytokine IL-12 were considered to be the only effector T cells inducing EAE. Paradoxically, however, mice deficient of either IFN-γ [1], IL-12 p35 subunit [2] or their corresponding receptors IFN-γR [3] and IL-12Rβ2 [4] were not protected, but highly susceptible to EAE induction. In contrast, mice treated with antibodies neutralizing the IL-12 p40 subunit, or mutant mice lacking IL-12 p40 subunit were resistant to EAE induction [5]–[8]. Cua et al. explained this paradox by the double usage of the IL-12 p40 subunit by both IL-12 and IL-23 (heterodimer of IL-12p40 and IL-23p19 subunits). In fact, this work demonstrated that IL-12 specific p35, but not IL-23 specific p19, is dispensable for EAE development [9]. IL-23 was shown to drive the maintenance and expansion of a distinct and newly identified CD4^+^ helper T cell subset, Th17 cells, which produced abundant amounts of IL-17 instead of IFN-γ [10].

Initially these findings seemed to suggest that Th17 cells but not Th1 cells were the only pathogenic effector cells in EAE. These conclusions were mainly drawn from studies of EAE actively induced by immunization with complete Freund's adjuvant, a harsh treatment that profoundly impacts the general immune response. More recently, however, studies of adoptive transfer EAE using polarized Th1 and Th17 cells support pathogenic roles for either subset. But some of the findings remained contradictory. O'Connor and colleagues demonstrated that MOG specific Th1 cells are highly pathogenic, and are required to facilitate entry of Th17 cells into CNS lesions [11]. Using MOG-specific transgenic T cells, Yang et al found that T-bet expression was essential for EAE induced by Th1 and Th17 cells [12]. Recently, another study proposed that the ratio of myelin-specific Th17 versus Th1 cells determines the site of CNS inflammation [13]. Similarly, both IL-12 and IL-23 driven myelin-reactive T cells were found to induce distinct clinical EAE outcomes [14]. Finally, in spontaneous mouse EAE models with different genetic backgrounds CNS lesions contained both Th1 and Th17 cells, suggesting that both T cell lineages participate in the autoimmune pathogenesis [15], [16].

In the present study, we searched for functional differences and pathogenic potential of the “monoclonal” MOG-specific CD4^+^ T cells with Th1- and Th17-like functional profiles. These were derived from MOG-specific TCR transgenic (2D2) mice with C57BL/6 genetic background. When adoptively transferred into lymphopenic hosts, either individually or combined, both Th1 and Th17 cells per se were capable of inducing EAE, but combinations of Th1 and Th17 cells displayed a potentiated effect. The clinical disease mediated by either CD4^+^ T cell lineage differed profoundly. While Th1 cells mediated classic EAE with hind limb paralysis, Th17 cells transferred a disease with ataxic gait in approximately half of the animals. Within the CNS infiltrates, Th17 cells seemed to convert to a Th1 phenotype, but not vice-versa. Finally, Th1 cells differed from Th17 cells by their cytotoxic potential. They lysed antigen presenting astrocytes in *in vitro* co-cultures, an activity not seen with Th17 cells.

## Results

### Differentiation and functional characterization of MOG-specific Th1 and Th17 cells

**Figure 1:**
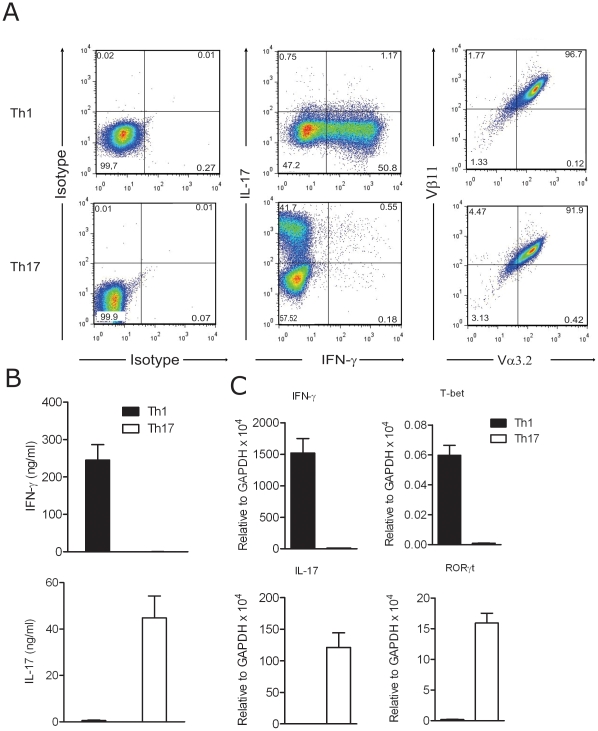
MOG-specific Th1 and Th17 cell differentiation. A. T cells from 2D2 mice were activated under Th1 and Th17 polarizing conditions. Vα3.2 and Vβ11 transgenic TCR chains, as well as intracellular IL-17 and IFN-γ cytokine expression was assessed by FACS. Data shown are gated in the CD4^+^ population. B. IFN-γ and IL-17 cytokines from culture supernatants of Th1 and Th17 polarized cells were quantified by ELISA. C. IFN-γ, Tbet, IL-17 and RORγt gene expression was quantified by real-time PCR of Th1 and Th17 polarized cells. Data shown are representative (A) or a mean of a minimum of 5 experiments. Error bars indicate SEM (B and C).

We used 2D2 TCR transgenic T cells that recognize MOG [17] to obtain sufficient numbers of Th1 and Th17 cells. We optimized *in vitro* differentiation protocols for Th1 and Th17 cells as described in methods. With this protocol, we obtained bulk numbers of Th1 and Th17 cells largely free of contaminating IL-17 and IFN-γ producing cells in Th1 and Th17 polarizations, respectively **(** **Figure 1** **)**. In Th1 cultures, we consistently obtained more than 50% of T cells that produced IFN-γ and in Th17 cultures 20–50% of cells produced IL-17. More than 90% of these cells expressed the Vα3.2/Vβ11 transgenic TCR chains **(** **Figure 1A** **)**. ELISA results confirmed that Th1 cells produced IFN-γ and Th17 cells produced large amounts of IL-17 in a mutually exclusive manner **(** **Figure 1B** **)**. Also, mRNA of the signature transcription factors for Th1 and Th17 cells, T-bet and RORγt respectively, together with IFN-γ and IL-17 were expressed selectively in their corresponding T cell subset **(** **Figure 1C** **)**.

**Figure 2:**
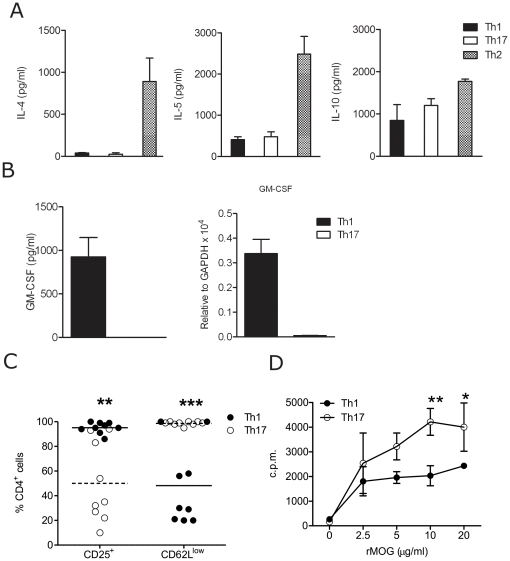
Comparative characterization of MOG-specific Th1, Th2 and Th17 cells. T cells from 2D2 mice were activated under Th1 and Th17 polarizing conditions. A. IL-4, IL-5, IL-10 and GM-CSF cytokines from culture supernatants of Th1, Th2 and Th17 polarized cells were quantified by ELISA (A and B). B. GM-CSF gene expression of Th1 and Th17 polarized cells was quantified by real-time PCR. C. CD25 and CD62L surface expression was assessed by FACS and represent the mean percentage of positive cells in the CD4^+^ population; **p<0.01; ***p<0.001. D. Antigen specific proliferation of differentiated Th1 and Th17 cells with titrated concentrations of rMOG was measured by quantification of radioactive ^3^H-thymidine uptake. *p<0.05; **p<0.01. Data shown are expressed as mean ± SEM of 3 (A, B), 9 (C) or representative of 3 (D) independent experiments.

To further understand the functional differences of Th1 and Th17 cells, we measured the expression of a panel of cytokines and activation markers. Both Th1 and Th17 cells did not produce appreciable levels of Th2-related cytokines IL-4 and IL-5 whereas they produced comparable amounts of anti-inflammatory cytokine IL-10 **(** **Figure 2A** **)**. GM-CSF, an important pro-inflammatory cytokine found to be important in EAE pathogenesis, was exclusively expressed by Th1 cells **(** **Figure 2B** **)**. The activation status of Th1 and Th17 cells was evaluated by flow cytometry. Th1 and Th17 cells markedly differed in the expression of the characteristic cell surface activation markers CD62L and CD25. While all Th17 cells were CD62L^low^, only about 50% of Th1 cells downregulated this receptor. On the other hand, Th17 cells did not express CD25, as did Th1 cells **(** **Figure 2C** **)**. Finally, the antigen-specific reactivity of MOG-specific polarized Th1 and Th17 cells was measured by a proliferation assay. Th17 cells exhibited a higher proliferative response than Th1 cells in response to their cognate antigen, MOG **(** **Figure 2D** **)**.

### Both Th1 and Th17 cells induce EAE, but with different clinical phenotype

**Figure 3:**
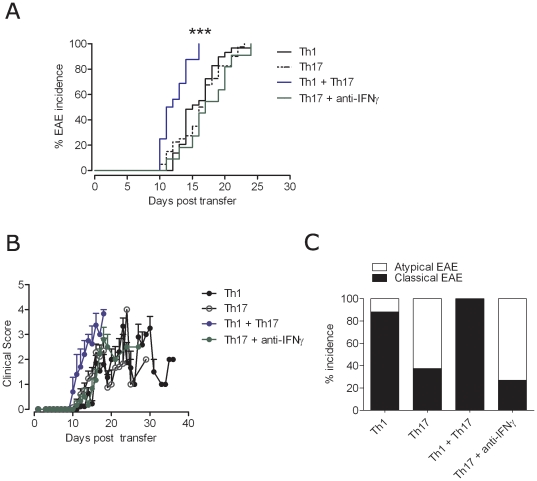
Adoptive transfer EAE with polarized Th1 and Th17 cells. Th1 and Th17 polarized cells were adoptively transferred to Rag2^−/−^ recipient mice, either alone or in combination or with anti-IFN-γ treatment as indicated. Recipient mice were scored for EAE disease. A. Shown is the percentage of EAE incidence in the different recipient mice. ***p<0.0001; Th1+Th17 vs. all other conditions. B. EAE clinical scores in the different recipient mice. Data is represented as mean ± SD. C. Incidence of classical and atypical EAE phenotype in different T cell transfers. Shown is the percentage of classical vs. atypical EAE incidence in the different recipient mice. Th1: n = 29; Th17: n = 40; Th1+Th17: n = 16; Th17+ anti-IFN-γ: n = 11.

We compared the encephalitogenic potential of MOG-specific Th1 and Th17 cell subsets in EAE by adoptive transfer. Three days after secondary *in vitro* stimulation in polarizing conditions, we transferred activated T cell blasts either individually or in combination into Rag2^−/−^ recipient mice. The use of lymphopenic recipients allowed us to evaluate the pathogenic potential of these CD4^+^ helper T cell subsets in the absence of host derived T and B cells. In this model, we observed 100% EAE incidence with similar day of disease onset, between 11 and 18 days post-transfer, in both Th1 and Th17 cells recipient animals. Interestingly, co-transfer of Th1 and Th17 cells induced EAE with earlier onset, between 10 and 13 days post-transfer, with severe disease (**Figures 3A, B**).

Th1 cells or Th1/Th17 co-transfers induced classical EAE in almost all recipients, characterized by a paralysis progressing from tail to head. In contrast, approximately 50% of Th17 cells recipients came down with an atypical neurological disease exhibiting an ataxia with an unbalanced gait and in few mice severe axial and barrel rotatory defects (**Figure 3C** **, Video S1**). Mice that recovered from such an ataxia eventually developed classical EAE symptoms such as paralysis. We compared CNS lesions of sick mice from all the adoptive transfer groups at the peak of the disease. Histological and immunohistochemistry analysis revealed that all groups exhibited severe immune cells infiltration (CD4^+^ T cells and macrophages), astrogliosis, microglia activation, demyelination and axonal damage. However, this was largely indistinguishable between Th1 and Th17 single transfers or between Th17 classic and ataxic EAE. Lesions were located throughout the CNS in both Th1 and Th17 recipients, with no significantly different preferential localization of CD4^+^ T cell infiltrates. In addition, we found infiltration and demyelination in the PNS, in particular the trigeminal root and the spinal roots in both groups **(** **Table 1** **, Figure S2 and data not shown)**.

**Table 1:** Quantification of inflammation and demyelination in spinal cord, brain and PNS.

Adoptive transfer EAE	EAE score	SC Inf	SC DM	Brain Inf	Brain DM	PNS
**Th1**	3.5	0.7	1	Cer, Obl, ON	Cer:1, Obl: 1, ON:0	TG
	3.5	0.5	1	Cer, Obl,	Cer 3; Obl 1,	TG
	3.5	1.9	2	Cer, Obl, ON	Cer 2, Obl 1, ON 1	TG
	4	2.1	1	ON	ON 1	TG, RO
	4	3.5	2	Cer, ON	Cer 1, ON 1	TG, RO
	4	2.8	2	Cer, Obl, Trig, ON	Cer 1, Obl 1, ON 1	TG, RO
**Th17**	4	2	2	Cer, obl	Cer 2	TG
	4	1.7	2	Cer, Obl, ON,	ON 1	TG, RO
	4	2.4	2	Cer, Obl, Trig	Trig 2	TG, RO
	ataxic	4.5	2	Cer, Obl, Trig, ON	Cer 2, Obl 1, Trig 3, ON 1	TG
	ataxic	1.3	1	Cer, Obl, Trig, ON	Cer 1, Obl 1, ON 2	TG, RO
**Th1+Th17**	4	2.2	3	Cer, Obl, ON,	ON 1	TG, RO
	4	2.2	2	Obl, Trig, ON	ON 1, Trig 2	TG, RO
	4	2	3	Cer, Obl, ON, Trig	ON 2, Trig 2	TG, RO

This quantification is according with the classification developed by [53]. SC Inf – inflammatory infiltrates per spinal cord section; SC DM – spinal cord extent of demyelination determined semi-quantitatively: 1-perivenous, 2-confluent, 3-profound (half of spinal cord section), 4-complete (entire spinal cord section); Brain Inf and Brain DM – Areas of the brain with inflammation and demyelination (with score as in SC): Cer-cerebellum, Obl-medulla oblongata, ON-optic nerves, Trig-central portion of the trigeminal root; PNS – inflammation in the peripheral nervous system: TG-trigeminal root, RO-spinal roots.

### Plasticity of Th17 cells *in vivo*

**Figure 4:**
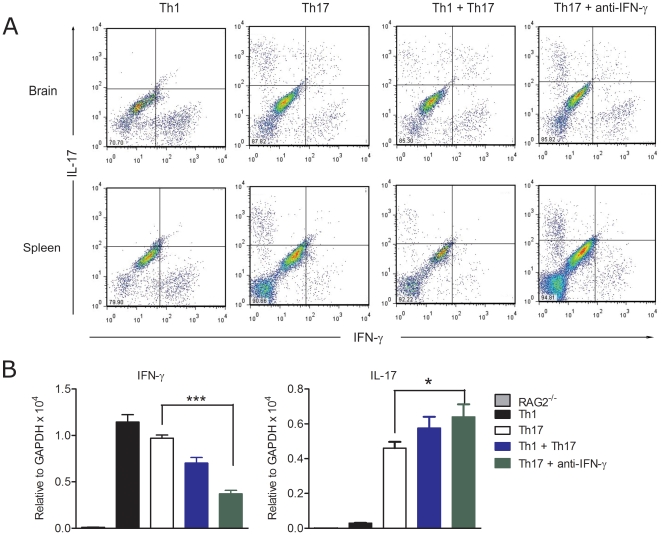
Plasticity of Th17 cells in adoptive transfer EAE. Th1, Th17, Th1+Th17 cells or Th17 cells and anti-IFN-γ antibodies were adoptively transferred into Rag2^−/−^ mice. A. EAE sick mice with classical EAE, minimum score of 3 were sacrificed, and brain and spleen were removed. Immune cells were isolated and characterized for IFN-γ and IL-17 expression by intracellular FACS staining. Data shown is gated in the CD4^+^ population and is representative of a minimum of 5 animals analyzed per group. B. Expression of IFN-γ and IL-17 in the brain from EAE sick mice was analyzed by real time PCR. Rag2^−/−^ control: n = 7; Th1: n = 7; Th17: n = 10; Th1+Th17: n = 5; Th17+anti-IFN-γ: n = 6. Data are represented as mean ± SEM. *p<0.05; ***p<0.0001.

We analyzed the cellular composition of CNS infiltrating mononuclear cells as well as peripheral lymphoid organs of Rag2^−/−^ recipients. Mice at the peak of paralytic or ataxic EAE had major infiltrates in both brain and spinal cord. Flow cytometric analysis for the intracellular cytokines showed that Th1 recipients contained mainly IFN-γ and negligible numbers of IL-17 producing CD4^+^ T cells in both brain and spleen. In contrast, mice with Th17 mediated EAE had both IFN-γ and IL-17 producing CD4^+^ T cells in similar proportions. In addition, we found IFN-γ^+^ IL-17^+^ double positive CD4^+^ T cell population in the CNS but not in the periphery of Th17 recipients (**Figure 4A**). Analysis of spinal cord and lymph nodes yielded similar results (data not shown).

The co-existence of IFN-γ^+^ and IFN-γ^+^ IL-17^+^ double positive cells in the CNS of Th17 recipient mice might suggest a possible conversion of Th17 cells to the Th1 phenotype or *de novo* induction from double negative cells *in vivo*. Since IFN-γ is a negative regulator of Th17 polarization, we hypothesized that IFN-γ produced by cells of the local milieu could have initiated this conversion. To neutralize such signals, we treated Th17 cell recipients with a blocking antibody for IFN-γ. However, such treatment had no effect on EAE onset or severity and majority of the treated mice developed atactic phenotype similar to untreated Th17 cells recipients **(** **Figure 3** **)**. More important, T cell conversion from Th17 to a Th1 phenotype was not impaired by blocking IFN-γ (**Figure 4A**). The expression levels of IFN-γ and IL-17 in the brains of Rag2^−/−^ recipients after transfer of Th1, Th17, mixture of Th1 and Th17 cells or Th17 cells and anti-IFN-γ blocking antibody were also measured. IL-17 was highly expressed in Th17 recipients whereas, in Th1 cells transfers, the levels were close to control. This is in agreement with flow cytometric analysis where IL-17 production was not observed in the brain of Th1 cells recipient mice. Expression levels of IFN-γ were higher in Th1 recipients. Nevertheless, the fact that IFN-γ was also expressed in Th17 recipient mice confirmed the phenotypic conversion of Th17 cells or *de novo* induction from double negative cells in the CNS. Neutralization of IFN-γ partially suppressed the IFN-γ expression while increasing IL-17 expression in the CNS **(** **Figure 4B** **)**. To determine whether Th1 and Th17 polarizing cytokines, such as IL-12 and IL-23 respectively, released by innate immune cells could have a role in Th17 cells plasticity, Th17 cells were transferred into Rag2^−/−^ × IL-12p35^−/−^ (deficient of IL-12) and Rag2^−/−^ × IL-12p40^−/−^ recipient mice (deficient of both IL-12 and IL-23). In both cases, IFN-γ production by the host would be compromised and phenotypic conversion might not happen. Contrary to our expectations, all these mice developed EAE albeit with delayed EAE onset and Th17 cells converted to IFN-γ producing cells (**Figure S1**).

### Cytotoxic ability of Th1 and Th17 cells towards astrocytes

During CNS autoimmunity, T cells that invade the CNS are thought to be reactivated by local antigen presenting cells such as astrocytes. Indeed, several studies suggest that astrocytes can present antigen to the invading T cells, and *in vitro* co-culture of astrocytes with activated myelin-specific T cells (primarily Th1 cells) can lyse those astrocytes presenting myelin peptides [18]. Further, highly activated astrocytes are common components of MS lesions and death of astrocytes can be unfavourable re-myelination processes.

**Figure 5:**
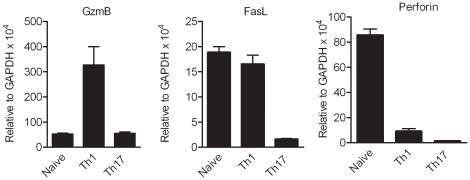
Cytotoxic molecules expression by Th1 and Th17 cells. T cells from 2D2 mice were activated under Th1 and Th17 polarizing conditions. Expression of granzyme B, Fas-L and perforin in naive T cells (n = 4), Th1 (n = 6) and Th17 cells (n = 7) were measured by real time PCR. The data is represented as mean ± SEM.

**Figure 6:**
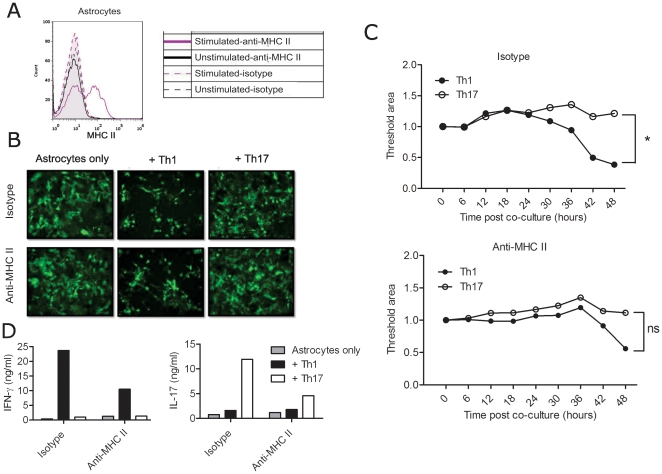
Cytotoxic potential of Th1 and Th17 cells towards astrocytes. 2D2 MOG-specific T cells were polarized in Th1 and Th17 conditions and co-cultured in duplicates with activated astrocytes as described in methods. A. MHC class II expression on astrocytes was analysed by FACS. B. GFP-labeled astrocytes were co-cultured with Th1 or Th17 cells in the presence of isotype control or anti-MHC class II antibodies. Cells were tracked every 30 minutes by fluorescent time-lapse microscopy. Shown is the snap-shot fluorescent picture after 48 h co-culture. Magnification: 10×. C. Quantification of the fluorescent area (surviving cells) every 6 hours in the conditions shown in B. The values were normalized to that of control (astrocytes only). D. IL-17 and IFN-γ levels were measured in the supernatants at 48 h after the co-culture by ELISA. Data shown are representative of minimum 3 experiments. *p<0.05; ns-not significant.

**Figure 7:**
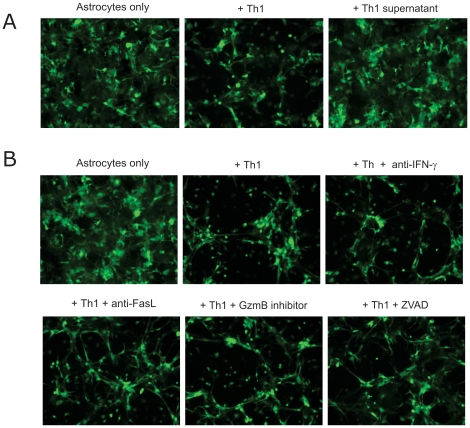
Effect of blockade of cytotoxic molecules on Th1 mediated astrocyte cytotoxicity. 2D2 MOG-specific Th1 cells were co-cultured in duplicates for 48 hours with astrocytes in the presence or absence of the Th1 culture supernatant (A), anti-IFN-γ antibody, anti-FasL antibody, Granzyme B (GzmB) inhibitor and the pan-caspase inhibitor ZVAD (B). Cells were tracked every 30 minutes by fluorescent time-lapse microscopy. Shown is the snap-shot fluorescent picture at the end of the culture period and is representative of minimum 2 experiments.

Th1 and Th17 cells, driven by IL-12 and IL-23 respectively, show distinct gene expression profile, which determine their functional capabilities. Microarray analysis of Th1 and Th17 cells by us (unpublished data) and others showed differential expression of cytotoxicity-related molecules in these two subsets [19]. In our *in vitro* polarized T cells, granzyme B was up-regulated in Th1 cells in relation to Th17 cells and naïve cells, and Fas-L expression was down-regulated in Th17 cells. Both Th1 and Th17 cells showed lower perforin expression than naïve T cells (**Figure 5**). Based on these findings, and to learn more about the differential pathogenic mechanisms mediated by Th1 and Th17 cells, we explored the cytotoxic potential of these distinct CD4^+^ T cell lineages. We investigated whether Th1 and Th17 cells possess the ability to lyse autoantigen-presenting astrocytes. Astrocytes were pre-treated with IFN-γ and TNF-α to up-regulate MHC class II expression on the surface **(** **Figure 6A** **)** and further co-cultured with T cells for 48 hours. We observed massive cytolysis of astrocytes induced by Th1 cells but not by Th17 cells. Blocking MHC class II only moderately suppressed the cytotoxicity by Th1 cells probably due to the modest up-regulation of MHC class II or by the highly activated nature of the T cells that were co-cultured **(** **Figures 6B, C** **Videos S2, S3)**. ELISA analysis of the co-culture supernatants showed that the extent of cytotoxicity might be correlated with the amount of IFN-γ produced by the Th1 cells, which is partially suppressed by MHC class II blocking **(** **Figure 6D** **)**. The addition of supernatant from the Th1 cultures, which contain abundant amounts of IFN-γ failed to induce cytolysis of astrocytes indicating a possible cell-to-cell contact mediated mechanism responsible for the cytotoxicity **(** **Figure 7A** **, Video S4)**. However, blocking IFN-γ did not prevent the cytotoxicity mediated by Th1 cells. Further, neither the anti-Fas-L antibody nor the inhibitors of granzyme B and pan-caspase (ZVAD) inhibitors prevented Th1-mediated astrocyte lysis **(** **Figure 7B** **, Videos S5, S6)**.

**Figure 8:**
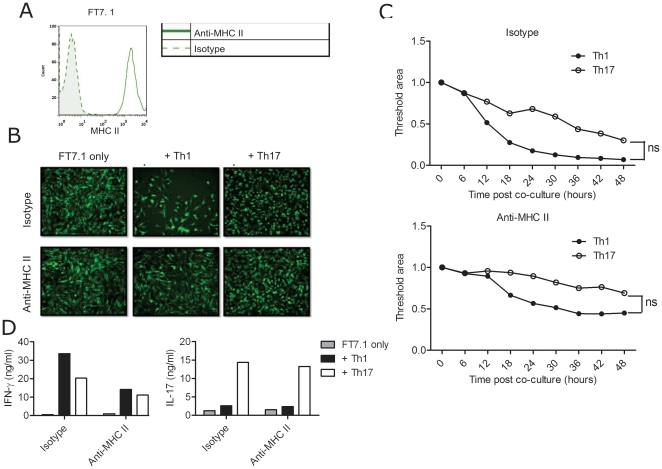
Cytotoxic potential of Th1 and Th17 cells towards FT7.1 cells. 2D2 MOG-specific T cells were polarized in Th1 and Th17 conditions and co-cultured in duplicates with FT7.1 cells as described in methods. A. MHC class II expression on FT7.1 cells was analysed by FACS. B. GFP-labeled FT7.1 cells were co-cultured with Th1 or Th17 cells in the presence of isotype control or anti-MHC class II antibodies. Cells were tracked every 30 minutes by fluorescent time-lapse microscopy. Shown is the snap-shot fluorescent picture after 48 h co-culture. Magnification: 10×. C. Quantification of the fluorescent area (surviving cells) every 6 hours in the conditions shown in B. The values were normalized to that of control (FT7.1 cells only). D. IL-17 and IFN-γ levels were measured in the supernatants at 48 h after the co-culture by ELISA. Data shown are representative of minimum 3 experiments. ns-not significant.

Since MHC class II expression was only moderately increased after stimulation, to evaluate the role of antigen presentation in Th1-mediated cytotoxicity, we performed T cell co-culture experiments with FT7.1 cells, a L cell fibroblast cell line stably transfected with the I-A^b^ molecule [20] **(** **Figure 8A** **)**. Again, we observed a massive cell death induced by Th1 cells and a moderate cytolysis by Th17 cells. MHC class II blocking almost completely prevented death of FT7.1cells in all cases **(** **Figure 8B, C** **, Video S7, S8)**. Intriguingly, Th17 cells also lysed FT7.1 cells albeit to a lower extent and delayed kinetics. Interestingly, quantification of IFN-γ and IL-17 in the co-culture supernatants revealed the presence of IFN-γ in addition to IL-17 in Th17 cells co-cultures suggesting a phenotypic conversion of Th17 cells to Th1 which might be responsible for the cytotoxicity (**Figure 8D** **)**. In addition, IFN-γ blocking partially prevented death of FT7.1 cells mediated by Th1 and Th17 cells (data not shown). Altogether, these data suggest a cell-to-cell contact dependent cytotoxic capacity of Th1 but not Th17 cells towards antigen presenting fibroblasts and astrocytes. Moreover, these data demonstrate that *in vitro* generated Th17 cells are plastic and convert to Th1 cells when recognize their cognate antigen, supporting our data about *in vivo* Th17 plasticity in our EAE model.

## Discussion

Depending on the major cytokines produced, CD4^+^ T cells have been classified into distinct subsets namely Th1, Th2, Th17, Th9, Tfh and Treg. Recently, numerous studies were undertaken to identify the T cell subset(s) primarily driving tissue-specific autoimmune diseases, such as EAE. They showed that, in principle, both Th1 and Th17 cells have the potential to mediate autoimmune pathogenesis, though through different pathological phenotypes [21]–[25].

Here, we compared the functional and pathogenic abilities of Th1 and Th17 helper CD4^+^ T cell subsets derived from TCR transgenic “monoclonal” T cells that recognize MOG and NF-M peptides in the context of I-A^b^. We used “monoclonal” T cells from 2D2 TCR transgenic mice to rule out functional variations due to different TCR repertoires. We optimized a protocol that allowed us to generate sufficient numbers and purity of these defined reactive cells and used them for adoptive transfer into Rag2-deficient mice. Our data agree with recent studies [24], [26] that both Th1 and Th17 cells alone are capable of mediating EAE. Furthermore, we show that Th1 and Th17 together synergize to enhance disease severity and pathology.

The polarized Th1 and Th17 cells produced abundant amounts of their signature cytokines, IFN-γ and IL-17 respectively. In addition, Th1 cells but not Th17 cells produced GM-CSF which is in agreement with a recent study [27]. Although IL-23 can induce the expression of GM-CSF in many innate cells, for unknown reasons, our defined culture conditions did not allow GM-CSF production by Th17 cells. Th1 but not Th17 cells readily upregulated the surface expression of CD25, the IL-2 receptor alpha chain commonly associated with the activation status of T cells. Recent studies suggest that IL-2 acts as a negative regulator of Th17 differentiation [28]. We speculate that Th17 cells do not upregulate CD25 to avoid any inhibition rendered by IL-2. Moreover, we added IL-23 but not IL-2 into our Th17 differentiation conditions which may further suppress CD25 expression.

The clinical EAE syndromes caused by Th1 and Th17 cells were notably distinct. While transfer of Th1 cells alone induced a classical EAE phenotype, characterized by an ascending caudocranial paralysis. In contrast, approximately half of the Th17 cells recipient mice developed atypical EAE with an ataxic gait disturbance, reminding of an EAE variant described by Stromnes et al in C3H mice immunized with MOG peptides. There, higher Th17 cell numbers led to inflammation in the brain and an atypical EAE, while Th1 skewing induced classic EAE [25]. Unexpectedly, the distinct clinical syndromes were not reflected by macroscopic distributions of the lesions, but there were histological distinctions. While lesions of Th1 recipients were dominated by Th1 cells, Th17 recipients had infiltrates composed of both IFN-γ and IL-17 producing T cells. We speculate that transient high local concentration of IL-17 in the brain might be responsible for the atypical EAE presentation. However, due to the emergence of IFN-γ producing T cells in Th17 recipients, lesions might spread throughout the CNS and remain indistinguishable between recipients leading to classical EAE. In fact, we observed that some Th17 recipients that presented ataxic disease progressed into classic paralytic EAE. Interestingly, all of the Th1+Th17 co-transfer mice developed only classical EAE demonstrating a dominant effect of IFN-γ on the disease outcome. This is in line with the previous finding that IFN-γ and its responsiveness of the CNS determine the lesion localization [29], [30].

The emergence of Th1-like cells in lesions initiated by transferred IL-17 producing Th17 cells could be explained by several mechanisms. Most prominently, Th1 cells could have emerged from the numerous neutral cells contained in the transferred inocula, or there might have been a shift from IL-17 to IFN-γ production. Previous studies of highly purified cells documented a high degree of plasticity of Th17 cells readily converting to Th1 phenotype *in vivo*, a process particularly prominent in the lymphopenic recipients [31], [32]. We used Rag2^−/−^ mice as recipients to avoid any host derived cells contributing to the pathogenesis [33] and this could have facilitated phenotype conversion. Converted IFN-γ producing Th1 cells were found both in the CNS and in the periphery and it equalled the numbers of IL-17 producing Th17 cells. Interestingly, in contrast to the previous studies [31], [32], we found IFN-γ^+^IL-17^+^ cells only in the CNS, not in peripheral lymphoid organs, suggesting a specific milieu or re-activation inside the CNS furthering the conversion to double cytokine producing T cells. Since host derived IFN-γ might antagonize the maintenance of IL-17 phenotype [34], we neutralized the IFN-γ *in vivo* by blocking antibodies. However, IFN-γ neutralization neither affected clinical disease nor phenotypic conversion of Th17 cells but partially suppressed IFN-γ related gene expression in the CNS. This could be due to the maximal activation of T cells before transfer or, more trivially, the exclusion of sufficient doses of antibodies from the CNS. *In vitro*, we observed conversion of Th17 cells to the Th1 phenotype only after renewed encounter with the antigen. Also, locally formed IL-12, the master cytokine inducing IFN-γ production in naïve CD4^+^ T cells, could have supported the conversion of Th17 cells [35]. However, unexpectedly, Th17 cells induced EAE in both IL-12p35 deficient (lacking IL-12 solely) and in IL-12p40 deficient mice (lacking both IL-12 and IL-23 and, in both hosts, we noted conversion of Th17 cells into IFN-γ producers. Recently it has been reported that a minor subset of Th17 cells can convert to IFN-γ producers in the absence of IL-12 *in vitro* [36]. We speculate that yet unknown factor(s), independent of IL-12, IL-23 might induce phenotypic conversion in vivo. Finally, it is possible that infiltrating Th17 cells or IFN-γ^−^IL-17^−^ cells that, in the CNS, local antigen-presenting cells such as astrocytes or microglia could have driven T cells from an IL-17 to IFN-γ production. Our data thus indicate a one-way phenotype conversion from IL-17- to IFN-γ producer but not vice-versa. The distinct clinical outcomes may be dependent on the extent of *in vivo* conversion of Th17 to the Th1 phenotype. In line with this, a recent report suggested that Th17 cells induced diabetes only upon conversion into IFN-γ producers *in vivo* [37].

Do Th1 and Th17 T cell subsets differ in their effector mechanisms? Distinct cytokine and chemokine expression by these cell types likely contribute to the differential migration and effector mechanisms at the target organ thus leading to different clinical outcomes. It is known that Th1 cells induce the expression of MHC class II or adhesion molecules on APCs by virtue of the production of IFN-γ, and thus facilitate the migration of T cells into CNS [38]–[41], activate macrophages [42] and induce Tregs [43]. On the other hand, Th17 cells producing IL-17A, IL-17F, IL-21 and IL-22, attract neutrophils to the site of inflammation [44], promote host defense [34] and regulate Tregs [45]. We found another principal difference between Th1 and Th17 effector T cells. Old work predating the identification of CD4^+^ T cell subsets discovered that the encephalitogenic T cells lyse myelin antigen presenting astrocytes [18], [46]–[48]. Indeed, we show here that our Th1 polarized cells lyse antigen presenting astrocytes and the fibroblasts. We used neutralizing antibodies to test the potential involvement of several lysis-related molecules (including IFN-γ MHC class II, granzyme B, caspases and FasL) in Th1-mediated cytotoxicity. None of them, with the exception of antibodies against MHC class II and IFN-γ, substantially blocked Th1 cell cytotoxicity. Whether this represents a yet unknown, cell-to-cell contact mediated mechanism potentiated by IFN-γ and antigen presentation remains to be shown. In contrast, Th17 cells failed to kill astrocytes and fibroblasts but acquired cytotoxic potential after converting to IFN-γ producing Th1 phenotype. Our future studies will address whether cytotoxic damage to astrocytes by Th1 autoimmune effector cells have a role in vivo. In addition to their supportive role in the CNS, astrocytes are activated in MS lesions and upregulate MHC class II to a certain degree [49]. Astrocyte targeted autoimmunity could also have a pathological consequence. Similar to neuromyelitis optica (NMO) where specific autoantibodies kill aquaporin-4 expressing astrocytes, a cytotoxic attack of myelin presenting astrocytes could contribute to the pathology in optic nerve and spinal cord of EAE and MS lesions [50].

## Materials and Methods

### Mice

2D2 (TCR^MOG^)[17], TCR^MOG^ × IgH^MOG^ double transgenic Optico-Spinal Encephalomyelitis (OSE) [15], Rag2^−/−^, IL-12p35^−/−^, IL-12p40^−/−^ and beta-actin GFP transgenic mice were bred in the animal facilities of the Max Planck Institute of Biochemistry and Neurobiology. All animals used in this study were with C57BL/6 background. The protocol was approved by the animal welfare committee of Government of Upper Bavaria (Tierschutzkommission der Regierung von Oberbayern, Munich, Germany) (License No: 55.2-1-54-2531-45/04). The animal procedures were in strict accordance with the guidelines set down by the animal welfare committee of the Government of Upper Bavaria.

### *In vitro* CD4^+^ T cell differentiation

Th1 and Th17 polarized cells were obtained after optimization of protocols described previously [51], [52]. Briefly, 20×10^6^ OSE or 2D2 total erythrocyte-lysed spleen cells per well were cultured for 6 days in the presence of 20 µg/ml rMOG (MOG aa1-125) in 6 well-plates in complete RPMI-1640 with 10% FCS. For Th1 and Th17 polarization, the following cytokines and antibodies were further added in the culture. Th1: IL-12 (10 ng/ml), IL-18 (25 ng/ml) and anti-IL-4 (10 µg/ml); Th17: human TGF-β1 (5 ng/ml), IL-6 (20 ng/ml), IL-23 (10 ng/ml), anti-IL-4 (10 µg/ml) and anti-IFN-γ (10 µg/ml); Th2: IL-4 (50 ng/ml) and anti-IFN-γ (10 µg/ml;. All cytokines were purchased from Peprotech except IL-23 (R&D Systems) and IL-18 (MBL). The neutralizing antibodies were produced from hybridoma supernatants. Th1 cells were supplemented with IL-2 (10 ng/ml, Peprotech) and Th17 cells with IL-23 (10 ng/ml) at day 3 of culture. 5–6 days later, living cells were purified by Nycoprep (Axis-Shield) gradient and CD4^+^ T cells were isolated by negative selection (R&D Systems) according to manufacturer's instructions. Purified CD4^+^ T cells (approximately 5×10^6^ cells/well) were polarized for a second time in the same conditions and in the presence of irradiated (30 Gy) splenic antigen presenting cells for additional 3 days. Activated living cells were purified by Nycoprep, yielding >99% pure CD4^+^ population.

### T cell proliferation assay

MOG-specific CD4^+^ T cells previously differentiated in Th1 and Th17 polarizing conditions were re-stimulated in triplicates (4×10^4^ T cells/well) in the presence of irradiated splenic cells (2×10^5^ cells/well) and rMOG for 72 h. Antigen specific T cell proliferation was measured by adding 1 µCi of [^3^H]-thymidine in the last 18 h and incorporated radioactivity was measured in the Beta counter.

### Adoptive transfer EAE

Freshly activated MOG-specific CD4^+^ Th1 and Th17 cells were suspended in PBS, counted and injected intravenously into Rag2^−/−^ recipient mice. Each animal received 5 to 10×10^6^ of Th1, Th17 or mixed Th1 and Th17 cells (proportion of 1∶2). In one group of Th17 cells transfer, anti-IFN-γ antibodies (500 µg/mouse) were injected at 4 days interval. Animals were evaluated every 1–2 days for clinical symptoms. The classical EAE scores were given as below: score 0 – no disease; score 0,5 – reduced tail tonus; score 1: limp tail; score 1,5 – limp tail and ataxia; score 2 – limp tail, ataxia and hind limb weakness; score 2,5 – at least one hind limb paralyzed/weakness; score 3 – both hind limbs paralyzed/weakness; score 3,5 –complete paralysis of hind limbs; score 4 – paralysis until hip; score 5 – moribund or dead. The non-classical (atypical) EAE scores were given as follows: score 0 – no disease; score 1 – head turned slightly (ataxia, no tail paralysis); score 2 – head turned more pronounced; score 3 – inability to walk on a straight line; score 4 – laying on side; score 4,5 – rolling continuously unless supported; score 5 – moribund or dead.

### Mononuclear cells isolation from CNS tissue

Sick mice were perfused transcardially with PBS. Spinal cord and brain tissue were isolated and homogenized in RPMI-1640 medium. Brain and spinal cord suspensions were passed through a 40 µm nylon mesh (BD Biosciences) and centrifuged. Cells were re-suspended in 30% percoll (GE Healthcare), overlaid on 70% Percoll and centrifuged for 20 min at 1200 g at room temperature. After centrifugation, the interface containing mononuclear cells was removed, washed with RPMI and used for flow cytometry.

### Flow cytometry

Single cell suspension of spleen and lymph nodes were prepared by nylon mesh. CNS infiltrating mononuclear cells were isolated by Percoll gradient. For intracellular cytokine staining, cells were stimulated for 4 hours with PMA (50 ng/ml), ionomycin (0.5 µg/ml) and brefeldin A (5 µg/ml) (Sigma Aldrich). Cells were first stained extracellularly in FACS buffer with fluorochrome labeled rat anti-mouse CD3e (145-2C11), CD4 (RM4-5), Vα3.2 TCR (RR3-16), Vβ11 (RR3-15), CD25 (PC61) and CD62L (MEL-14) antibodies (all from BD Pharmingen except CD62L, from Immuno Tools). Stained cells were washed, then fixed and permeabilized with 2% PFA and saponin buffer and finally stained intracellularly with anti-IFN-γ (XMG1.2) and anti-IL-17 (TC11-18H10) (BD Pharmingen) and their respective isotype controls in saponin buffer. Astrocytes and FT7.1 cells were stained with anti-MHC class II (2G9). Samples were acquired on a FACSCalibur (BD Biosciences) and data were analysed with CellQuest (BD Bioscience) and Flow Jo version 7.2.5 (Tree Star) softwares.

### Enzyme linked immunosorbent assay (ELISA)

Cytokine production was determined by ELISA using matching antibody pairs for IFN-γ (purified capture antibody clone AN-18, biotinylated detection antibody clone XMG1.2, BD Pharmingen), IL-17 (purified capture antibody clone eBio17CK15A5, biotinylated detection antibody clone eBio17B7, eBioscience), IL-4 (purified capture antibody clone BVD4-1D11, biotinylated detection antibody clone BVD6-24G2, BD Pharmingen), IL-5 (purified capture antibody clone TRFK5, biotinylated detection antibody clone TRFK4, BD Pharmingen), IL-10 (R&D Systems) and GM-CSF (Peprotech) according to the manufacturer's instructions. Culture supernatants were collected and frozen at −20°C until quantification.

### Quantitative real-time PCR analysis

Total RNA was isolated with TRI Reagent extraction (Sigma-Aldrich). 1–4 µg of RNA was treated with DNase I and then reverse transcribed into cDNA using oligo-dT primers and SuperScript II Reverse Transcriptase (Invitrogen), according to manufacturer's instructions. Primers and probes **(Table S1)** (Metabion, Martinsried, Germany) were used for SYBR Green or TaqMan PCR analysis. Where possible, the primer/probe sequence combinations spanned contact sequences of subsequent exons. For amplification, the Absolute QPCR mix was used (ABgene). Each reaction was run in triplicate on an ABI 7900 machine (Applied Biosystems) and was normalized to housekeeping gene GAPDH transcripts. Primary data was analyzed with Gene-Amp SDS version 2.3 software (Applied Biosystems).

### Histological analysis

Animals were perfused with cold PBS and then with 4% paraformaldehyde in PBS, stored in the same fixative for 24 hours at 4°C, washed twice with PBS, and finally kept at 4°C until used. Brain and spinal cord tissue was dissected and in part embedded in paraffin, or snap frozen in Tissue Tek OCT compound on dry ice for immunohistochemistry. Adjacent serial sections were stained with hematoxylin (H&E), luxol fast blue (LFB), or Bielschowsky silver impregnation (Biel).

### Astrocytes primary culture

Primary astrocyte cell cultures were obtained from 2-days-old beta-actin GFP transgenic or wild type C57BL/6 mouse pups. Briefly, brains were removed, placed in 15 mM of Hepes in Hanks Balanced Salt Solution (HBSS) (Gibco) and meninges were removed. Brains were homogenized in 15 mM of Hepes in HBSS with 1 ml tip and a 27 G syringe and cells were dissociated with incubation at 37°C for 10 minutes in 2 mg/ml of trypsin solution. After washing, cells were suspended in supplemented DMEM (Gibco), passed through a 70 µm cell strainer, and plated in a T75 flask. Cells were allowed to grow for 8 to 10 days and media was changed every 3 days. Cells were shaken overnight at 90 rpm to remove contaminating oligodendrocytes, microglia and neurons. Adherent cells contain a majority of astrocytes whose purity was increased with trypsinization and further passages. Experiments were done with astrocytes with a minimum of 2–3 passages.

### FT7.1 cell line culture

The fibroblast cell transfectants FT7.1, which overexpress the mouse I-A^b^ MHC class II molecule on their surface, were cultured in supplemented RPMI media plus the selective reagents: mycophenolic acid (2.5 mg/100 ml), xanthine (25 mg/100 ml) and hypoxanthine (2.5 mg/100 ml) (Sigma). For the use in time-lapse fluorescent microscopy experiments, FT7.1 cells were retrovirally transduced with GFP (pMSCVneo-IRES2-eGFP).

### T cells and astrocytes or FT7.1 cells co-culture

2 days before the co-culture, primary GFP-astrocytes were trypsinised, irradiated with 30 Gy and plated 4×10^4^ cells per well in 96-well plate. On the following day, adherent astrocytes were stimulated with IFN-γ and TNF-α, 10 ng/ml each (Peprotech).GFP-FT7.1 cells were plated (2.5×10^4^ cells per well) in 96-well plate. Freshly activated T cells were added to stimulated astrocytes or FT7.1 cells in a ratio of 1∶10, in the presence of 20 µg/ml MOG 35–55 peptide. The following blocking antibodies and inhibitors were used: 10 µg/ml anti-MHC class II (2G9), 10 µg/ml anti-IFN-γ (R4-6A2), 10 µg/ml anti-Fas-L, 10 µg/ml isotype control IgG2a (all from BD Pharmingen), 25 µM Granzyme B inhibitor II (Calbiochem) and 25 µM caspase inhibitor Z-VAD (Calbiochem). Cytotoxicity was evaluated by fluorescent time-lapse microscopy for 48 h using the MetaMorph software (Molecular Devices). For quantification, area covered by the fluorescent intact cells was calculated after background correction for each image using ImageJ. The data were normalized to that of control for each time point.

### Statistics

Descriptive statistical analysis was performed using Prism version 5 software (GraphPad). Differential EAE incidence was analyzed by log-rank (Mantel-Cox) test by an in-built survival curve analysis. One- and two-way ANOVA and t test statistical analysis were used in the other studies. p values less than 0.05 were considered to be significant.

## Supporting Information

Figure S1 **Adoptive transfer EAE of polarized Th17 cells in mice deficient of IL-12p35 or IL-12p40.** A. *In vitro* polarized Th17 cells were adoptively transferred to Rag2^−/−^, Rag2^−/−^ × IL-12p35^−/−^ and Rag2^−/−^ × IL-12p40^−/−^ mice as described in methods. Recipient mice were scored for EAE disease. Shown is the evaluation of percentage of EAE incidence in the different recipient mice. *p<0.001 in Rag2^−/−^ vs. other conditions. n = 5 animals per group. B. CNS infiltrating cells were isolated from sick mice of the above adoptive transfer and characterized for IFN-γ and IL-17 expression by intracellular FACS staining. Data shown is gated in the CD4^+^ population.
(TIF)

Figure S2 **Histological analysis of Th1 and Th17 cells recipient mice.** Spinal cord sections of Rag2^−/−^ recipient mice were characterized by histology and immunohistochemistry. H&E – haematoxylin & eosin, LFB – Luxol Fast Blue, Biel – Bielschowsky, CD3- for T cells, and MAC3 is a marker for phagocytes. Magnification: 25×. Representative figures are shown. Th1: n = 6; Th17: n = 5; Th1+Th17: n = 3.
(TIF)

Video S1 **Different EAE clinical phenotypes induced by Th1 and Th17 cells.** Shown is Th1 cells recipient mice with classical EAE, score 3 - tail and both hind limbs paralyzed (left); and Th17 cells recipient mice with atypical EAE, score 3.5 - inability to walk on a straight line and laying on side (right).
(WMV)

Video S2 **Th1 but not Th17 cells lyse astrocytes.** GFP-positive astrocytes were co-cultured with Th1 or Th17 cells for 48 h in the presence of isotype control antibody. Cells were tracked every 30 minutes by fluorescent time-lapse microscopy.
(AVI)

Video S3 **Th1 but not Th17 cells lyse astrocytes.** GFP-positive astrocytes were co-cultured with Th1 or Th17 cells for 48 h in the presence of MHC class II blocking antibody. Cells were tracked every 30 minutes by fluorescent time-lapse microscopy.
(AVI)

Video S4 **Effect of blockade of cytotoxic molecules on Th1 mediated astrocyte cytotoxicity.** GFP-positive astrocytes were co-cultured with Th1 cells or Th1cell culture supernatants for 48 h. Cells were tracked every 30 minutes by fluorescent time-lapse microscopy.
(AVI)

Video S5 **Effect of blockade of cytotoxic molecules on Th1 mediated astrocyte cytotoxicity.** GFP-positive astrocytes were co-cultured with Th1 cells with and without the presence of anti-IFN-γ antibody for 48 h. Cells were tracked every 30 minutes by fluorescent time-lapse microscopy.
(AVI)

Video S6 **Effect of blockade of cytotoxic molecules on Th1 mediated astrocyte cytotoxicity.** GFP-positive astrocytes were co-cultured with Th1 cells in the presence of anti-FasL antibody, Granzyme B (GzmB) inhibitor or the pan-caspase inhibitor ZVAD for 48 h. Cells were tracked every 30 minutes by fluorescent time-lapse microscopy.
(AVI)

Video S7 **Cytotoxic potential of Th1 and Th17 cells towards FT7.1 cells.** MHC class II overexpressing GFP-positive FT7.1 cells were co-cultured with Th1 or Th17 cells for 48 h in the presence of isotype control antibody. Cells were tracked every 30 minutes by fluorescent time-lapse microscopy.
(AVI)

Video S8 **Cytotoxic potential of Th1 and Th17 cells towards FT7.1 cells.** MHC class II overexpressing GFP-positive FT7.1 cells were co-cultured with Th1 or Th17 cells for 48 h in the presence of MHC class II blocking antibody. Cells were tracked every 30 minutes by fluorescent time-lapse microscopy.
(AVI)

Table S1 **Primers and probes used for real-time PCR.**
(DOC)
