# Supplementary material for: Functional and Pathogenic Differences of Th1 and Th17 Cells in Experimental Autoimmune Encephalomyelitis
Source: PLoS One. 2010 Nov 29;5(11):e15531. doi: 10.1371/journal.pone.0015531 (PMC3000428; doi:10.1371/journal.pone.0015531)
Supplement: Table S1 — Primers and probes used for real-time PCR. (DOC) [file pone.0015531.s011.doc]

**Supplemental table:**

**Table S1**

Primers and probes used for real-time PCR

| **Primers and probes** | **Primer name** | **Oligo sequence (5' -> 3')** | **References** |
| --- | --- | --- | --- |
| Fas-L | Fas-L sense | GAAGGAACTGGCAGAACTCCG | [1] |
|  | Fas-L anti-sense | CCCTGTTAAATGGGCCACACT |  |
|  | Fas-L probe | AAAGCAAATAGCCAACCCCAGCACACC |  |
| GAPDH | GAPDH sense | TCACCACCATGGAGAAGGC | Designed by Primer 3 software |
|  | GAPDH anti-sense | GCTAAGCAGTTGGTGGTGCA |  |
|  | GAPDH probe | ATGCCCCCATGTTTGTGATGGGTGT |  |
| GM-CSF | GM-CSF sense | GCCATCAAAGAAGCCCTGAA | [2] |
|  | GM-CSF anti-sense | GCGGGTCTGCACACATGTTA |  |
|  | GM-CSF probe | ACATGCCTGTCACATTGAATGAAGAGGTAGAAG |  |
| Granzyme B | GzmB sense | CGATCAAGGATCAGCAGCCT | [1] |
|  | GzmB anti-sense | CTTGCTGGGTCTTCTCCTGTTCT |  |
|  | GzmB probe | TGCTGCTCACTGTGAAGGAAGTATAATAAATGTCACT |  |
| IFN−γ | IFN−γ sense | TCAAGTGGCATAGATGTGGAAGAA | [3] |
|  | IFN−γ anti-sense | TGGCTCTGCAGGATTTTCATG |  |
|  | IFN−γ probe | TCACCATCCTTTTGCCAGTTCCTCCAG |  |
| IL-17A | IL-17 sense | AACTCCCTTGGCGCAAAAGT | Designed by Primer 3 software |
|  | IL-17 anti-sense | GGCACTGAGCTTCCCAGATC |  |
|  | IL-17 probe | CCACGTCACCCTGGACTCTCCACC |  |
| Perforin | Perforin sense | GAAGACCTATCAGGACCAGTACAACTT | [1] |
|  | Perforin anti-sense | CAAGGTGGAGTGGAGGTTTTTG |  |
|  | Perforin probe | ACCAGGCGAAAACTGTACATGCGACACT |  |
| RORγt | RORγt sense | CCGCTGAGAGGGCTTCAC | [4] |
|  | RORγt anti-sense | TGCAGGAGTAGGCCACATTACA |  |
|  | RORγt probe | AAGGGCTTCTTCCGCCGCAGCCAGCAG |  |
| T-bet | T-bet sense | GCCAGGGAACCGCTTATATG | [5] |
|  | T-bet anti-sense | GACGATCATCTGGGTCACATTGT |  |
|  | T-bet probe | ACCCAGACTCCCCCAACACCGG |  |

Reference List

1. Halloran PF, Urmson J, Ramassar V, Melk A, Zhu LF et al. (2004) Lesions of T-cell-mediated kidney allograft rejection in mice do not require perforin or granzymes A and B. Am J Transplant 4: 705-712. 10.1111/j.1600-6143.2004.00421.x [doi];AJT421 [pii].

2. Overbergh L, Giulietti A, Valckx D, Decallonne R, Bouillon R et al. (2003) The use of real-time reverse transcriptase PCR for the quantification of cytokine gene expression. J Biomol Tech 14: 33-43.

3. Giulietti A, Overbergh L, Valckx D, Decallonne B, Bouillon R et al. (2001) An overview of real-time quantitative PCR: applications to quantify cytokine gene expression. Methods 25: 386-401. 10.1006/meth.2001.1261 [doi];S1046-2023(01)91261-7 [pii].

4. Ivanov II, McKenzie BS, Zhou L, Tadokoro CE, Lepelley A et al. (2006) The orphan nuclear receptor RORgammat directs the differentiation program of proinflammatory IL-17+ T helper cells. Cell 126: 1121-1133. S0092-8674(06)01105-6 [pii];10.1016/j.cell.2006.07.035 [doi].

5. Lighvani AA, Frucht DM, Jankovic D, Yamane H, Aliberti J et al. (2001) T-bet is rapidly induced by interferon-gamma in lymphoid and myeloid cells. Proc Natl Acad Sci U S A 98: 15137-15142. 10.1073/pnas.261570598 [doi];98/26/15137 [pii].
